# Cerebrospinal fluid and serum cytokine profiles in severe viral encephalitis with implications for refractory status epilepticus: a retrospective observational study

**DOI:** 10.3389/fimmu.2025.1528763

**Published:** 2025-02-10

**Authors:** Peipei Huang, Fan Yang, Ruirui Dong, Lijun Wen, Qiuling Zang, Dandan Song, Junshuang Guo, Yating Wang, Ruike Zhang, Zhiping Ren, Jinjin Qin, Junfang Teng, Wang Miao

**Affiliations:** Neurological Intensive Care Unit, The First Affiliated Hospital of Zhengzhou University, Zhengzhou, China

**Keywords:** cerebrospinal fluid, cytokines, serum, severe viral encephalitis, refractory status epilepticus

## Abstract

**Background:**

To identify new intervention targets, we explored the correlation between cytokines and the development of refractory status epilepticus (RSE) in patients with severe viral encephalitis (SVE).

**Methods:**

We examined the characteristics of 14 cytokines in the cerebrospinal fluid (CSF) and serum, analyzing their correlation with acute symptomatic seizures and prognosis. Furthermore, we conducted a dynamic analysis of differences and correlations in the expression of cytokines among patients with SVE without seizures, those with controlled seizures, and those with RSE.

**Results:**

We included 161 patients with SVE; the incidence of seizures was 55.2%, and the mortality rate was 5.5%. Notably, 18.9% of these patients developed RSE, with a mortality rate of 20%. During the early stage of SVE, CSF interleukin (IL)-6 and IL-8 levels were significantly higher, declining over time and affecting the prognosis. CSF IL-6 and IL-8 levels were significantly elevated in the RSE group compared to patients without seizures and with controlled seizures, decreasing gradually and independently of serum cytokine levels. CSF IL-8 and age were independent risk factors for RSE, with clinical utility.

**Conclusions:**

Patients with SVE exhibit intrathecal cytokine storms, primarily characterized by elevated levels of IL-6 and IL-8, which influence prognosis. The strong and persistent hyperinflammation underscored by CSF IL-6 and IL-8 is associated with the occurrence and development of RSE; thus, CSF IL-8 and age are independent risk factors for SVE with RSE, indicating potential anti-inflammatory intervention targets.

## Graphical Abstract

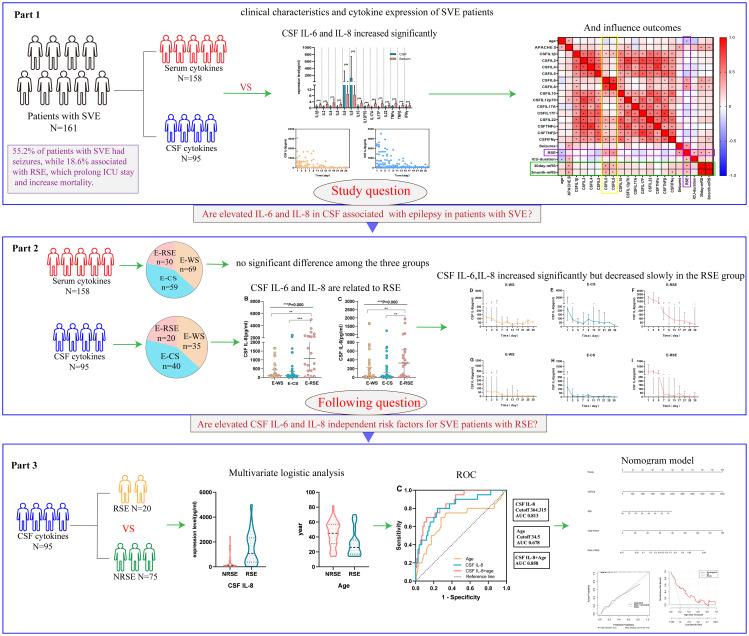

## 1 Introduction

Severe viral encephalitis (SVE) is usually characterized by increased intracranial pressure, delirium, coma, status epilepticus (SE), and respiratory failure. Patients with this condition are prone to complications, requiring treatment in the intensive care unit (ICU). Refractory status epilepticus (RSE) or super-refractory status epilepticus (SRSE) is a major factor affecting the prognosis and mortality of SVE (1). RSE occurs when sufficient doses of first-line antiseizure medications (ASMs), such as benzodiazepines, are administered, but the addition of another ASM fails to terminate the convulsive seizures and electroencephalogram epileptiform discharges (2). SRSE is a condition where treatment of SE with anesthetics exceeds 24 h, including the time during which the maintenance or tapering of the anesthetic and clinical convulsive seizures and electroencephalogram epileptiform discharges remain uncontrollable or recur (2, 3). Previous studies revealed 1-year mortality rates of 25.4% and 40% for patients with RSE and SRSE, respectively, in the intensive care unit (ICU) (4, 5). Therefore, identifying the factors associated with SVE complicated by RSE is urgent.

The inflammation induced by cytokines is closely associated with epileptogenesis. Previous studies have indicated that microglia, astrocytes, and infiltrating macrophages are potential cellular dynamics of new-onset RSE (6). Higher concentrations of cytokines can activate astrocytes or microglia in the central nervous system, leading to the abnormal release of excitatory neurotransmitters and triggering seizures (7).These high concentrations are also associated with a persistent state of seizures, often leading to uncontrolled and repeated epileptic seizures (8, 9). Studies have demonstrated elevated cerebrospinal fluid (CSF) cytokines (CSF-CKs) and serum cytokines (S-CKs) levels in viral encephalitis (10, 11). However, their relevance and whether elevated cytokine levels are associated with SVE complicated by acute symptomatic seizures or RSE remain unclear. Therefore, monitoring peripheral and central cytokine levels and analyzing their correlation with acute symptomatic seizures and RSE may help to elaborate the mechanisms underlying epileptogenesis and the development of seizures in patients with SVE.

Additionally, the factors influencing epilepsy are complex and diverse, and current research suggests that multiple ion channels are involved in epileptogenesis (12).T lymphocytes play a key role in the early onset of seizures in autoimmune epilepsy (13). Thyroid hormones play essential roles in the pathology of epilepsy through excitatory/inhibitory imbalance and influence several genes involved in its development (14). Studies on factors playing crucial roles in development of SVE complicated by RSE are limited; therefore, identifying risk factors for this condition is the basis for the clinical realization of active interventions.

This study aimed to analyze the clinical data, CSF-CKs, and S-CKs of patients with SVE to explore the correlation between cytokines, acute symptomatic seizures, and RSE. Additionally, the risk factors for RSE were analyzed to identify possible anti-inflammatory intervention targets for patients with SVE complicated by RSE.

## 2 Methods

### 2.1 Study population and design

We searched for “viral encephalitis,” “viral meningitis,” and “viral meningoencephalitis” in the patient database of the First Affiliated Hospital of Zhengzhou University from January 2020 to August 2023 and identified 1,050 patients. Among these, 229 met the criteria for SVE. First, we evaluated all patients for satisfaction of the 2013 diagnostic criteria for encephalitis (15), and ensured that their medical history, examination findings, CSF cytology, and protein and glucose assessments aligned with the changes recommended by the 2010 European Federation of Neurological Societies (EFNS) diagnostic methods for viral meningoencephalitis (16). Then, patients with a combination of one of the following conditions and an ICU admission for >48 h (17) were diagnosed with SVE: (i) respiratory failure requiring mechanical ventilation, (ii) mental behavioral abnormalities requiring sedatives, (iii) loss of alertness and. (iv)severe intracranial hypertension. The inclusion criteria were as follows: (i) age ≥14 and <80 years, (ii) S-CKs or CSF-CKs testing conducted after admission, and (iii) early stage of onset (onset time <2 weeks). The exclusion criteria were as follows: (i) patients with bacterial, tuberculous, or fungal encephalitis, meningitis, encephalomyelitis, or autoimmune encephalitis; (ii) patients with a combination of intracranial hemorrhage, cerebral infarction, or intracranial tumor; or (iii) patients with incomplete clinical data. Ultimately, 161 patients with SVE were enrolled.

### 2.2 Standard protocol approvals, registrations, and patient consent

This study was approved by the Ethics Committee of the First Affiliated Hospital of Zhengzhou University (Approval No. 2023-KY-0375-001). The requirement for informed consent was waived according to the national legislation and institutional requirements.

The patients with SVE were categorized into three groups as follows: encephalitis without seizures (E-WS; this included patients with viral encephalitis but no seizures), encephalitis with controlled seizures (E-CS; patients with acute symptomatic seizures that quickly terminated without recurrence and did not meet the diagnostic criteria for RSE), and encephalitis with RSE (E-RSE; patients clinically diagnosed with RSE [including SRSE]). All types met their respective defined criteria (2).

### 2.3 Data collection

Patients’ clinical data were collected, including age, sex, severity at admission (assessed using the Glasgow Coma Scale score and Acute Physiology and Chronic Health Evaluation-II), inducement, clinical symptoms, imaging results, duration of ICU hospitalization, mechanical ventilation, and ASM administration. CSF data were collected, including CSF pressure, CSF cytology, CSF biochemistry, albumin quotient, CSF-CKs levels, and etiology. Additionally, S-CKs, complete blood count, procalcitonin, electrolytes, thyroid function, lymphocyte subsets, and other data were collected. Patients received outpatient visits or telephone follow-ups at 30 days and 3 months, and modified Rankin Scale assessment was performed, with a score of 0–2 suggesting a favorable prognosis and 3–6 indicating a poor prognosis.

On the second morning after the patients’ admission, 3 mL of whole blood was collected using a procoagulant tube. The samples were left to clot for 30 min at room temperature and then centrifuged at 1,000–1,200 ×*g* for 15 min to obtain the serum for testing. Lumbar punctures were performed within 24 h of admission to collect 2 mL of CSF samples. Dynamic analysis was performed based on the results of regular re-examinations of CSF-CKs and S-CKs. We utilized AimPlex Multiple Immunoassays for Flow (Tianjin Kuangbo Tongsheng Biotechnology Co., Ltd.) to detect the levels of 14 cytokines in the serum and CSF. These cytokines included interleukin (IL)-1β, IL-2, IL-4, IL-5, IL-6, IL-8, IL-10, IL-12P70, IL-17A, IL-17F, IL-22, tumor necrosis factor (TNF)-α, TNF-β, and IFN-γ. The concentrations of all cytokines are expressed in pg/mL.

### 2.4 Statistical analysis

Normality of clinical sign variables and cytokines distribution in patients with SVE was assessed using the Shapiro–Wilk test (n<;50 samples) and Kolmogorov-Smirnov test (n≥50 samples). Normally expressed data are shown as mean ± standard deviation; otherwise, they are expressed as median (interquartile range [IQR]). The Mann–Whitney U test was used for comparison of non-normally distributed data. A heatmap of the Spearman’s correlation coefficient (r value) related to prognosis was created.

Among the three groups (E-WS, E-CS, and E-RSE), the Hartley test was used to assess the homogeneity of variance. As the variances were identified to be heterogeneous, the Kruskal–Wallis test was used to analyze differences, followed by *post-hoc* comparisons among the groups with significant differences.

Univariate logistic regression analyses were used to screen for risk variables associated with SVE complicated by RSE. Variables significantly associated with RSE in the univariate analysis were included in the multivariate logistic regression analysis. The relationship between risk variables and RSE were visualized using receiver operating characteristic (ROC) curves. In addition, we developed a nomogram to illustrate the risk factors for RSE and evaluated the clinical value of the model using the Hosmer–Lemeshow test and decision curve analysis. Data analysis was performed using SPSS 25.0 (IBM, Armonk, NY), and the graphs were generated with GraphPad Prism 8.0 (GraphPad Software, San Diego, CA) and R software4.2.2 (R Foundation, Vienna, Austria).

## 3 Results

### 3.1 Clinical characteristics and cytokine expression in SVE

#### 3.1.1 Clinical characteristics of SVE

In total, 161 patients with SVE were included, with an average age of 42 ± 18 years. Males accounted for 59% of the study population. The median duration of ICU stay was 10 days (IQR: 6–17). Moreover, 55.2% (89/161) had seizures, 40.3% (65/161) required mechanical ventilation, and the mortality rate was 5.5% (9/161). Additionally, 18.6% (30/161) of patients had comorbid RSE, 40% (12/30) of whom progressed to SRSE and 66.6% (20/30) of whom required mechanical ventilation. The median ICU stay for patients with RSE was 17 days (IQR: 8–28), and the mortality rate was 20% (6/30) (**Supplementary Table 1**).

#### 3.1.2 Comparative analysis of CSF-CKs and S-CKs in SVE

**Figure 1:**
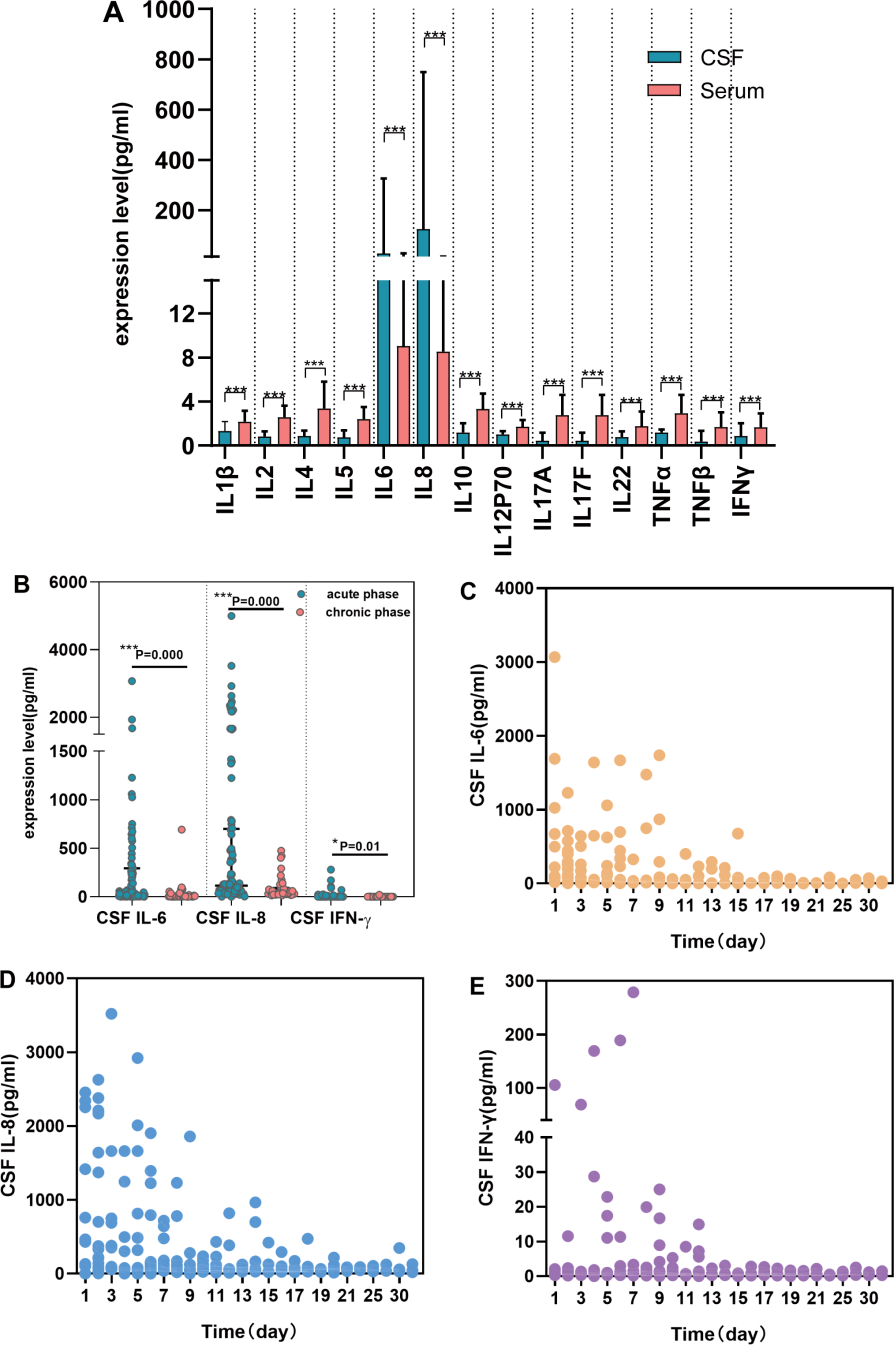
Comparative analysis of CSF and serum cytokines and changes over time in SVE. Comparison of cytokines’ expression in CSF (n = 95) and serum (n = 158) in SVE **(A)**(P<;0.001). Expression characteristics of CSF cytokines over time in SVE (n = 95) **(B-E)**. CSF IL-6, CSF IL-8, and CSF IFN-γ were elevated during the acute phase **(B)** (P<;0.05). The trend of CSF IL-6 **(C)**, CSF IL-8 **(D)** and CSF IFN-γ **(E)** over time respectively (***P < 0.001, *P < 0.05). CSF, cerebrospinal fluid; SVE, severe viral encephalitis; TNF, tumor necrosis factor; IFN, interferon; IL, interleukin.

A comparative analysis between S-CKs (n = 158) and CSF-CKs (n = 95) revealed significantly elevated levels of IL-6 with a median of 28.55 mg/dL (IQR: 5.71–326.1) and IL-8 with a median of 125.35 mg/dL (IQR: 59.00–748.90) in the CSF, contrary to the corresponding S-CKs, with significant differences (P < 0.001). The remaining 12 CSF-CKs were lower than their serum counterparts (P < 0.001) (**Figure 1A**, **Supplementary Table 2**).

#### 3.1.3 Characterization of cytokine changes over time in the CSF and serum in SVE

CSF and serum cytokine analyses in patients with SVE within 24 h of admission showed markedly elevated CSF IL-6 and IL-8 levels (P < 0.001) as well as increased IFN-γ levels (P = 0.015, **Figure 1B**). The trend chart of CSF IL-6, IL-8, and IFN-γ levels over time showed an overall downward trend with time, decreasing to the baseline level after 2 weeks (**Figures 1C-E**). No significant changes in S-CKs levels were observed over time.

#### 3.1.4 Spearman’s correlation coefficients between cytokine levels, RSE, and outcomes

**Figure 2:**
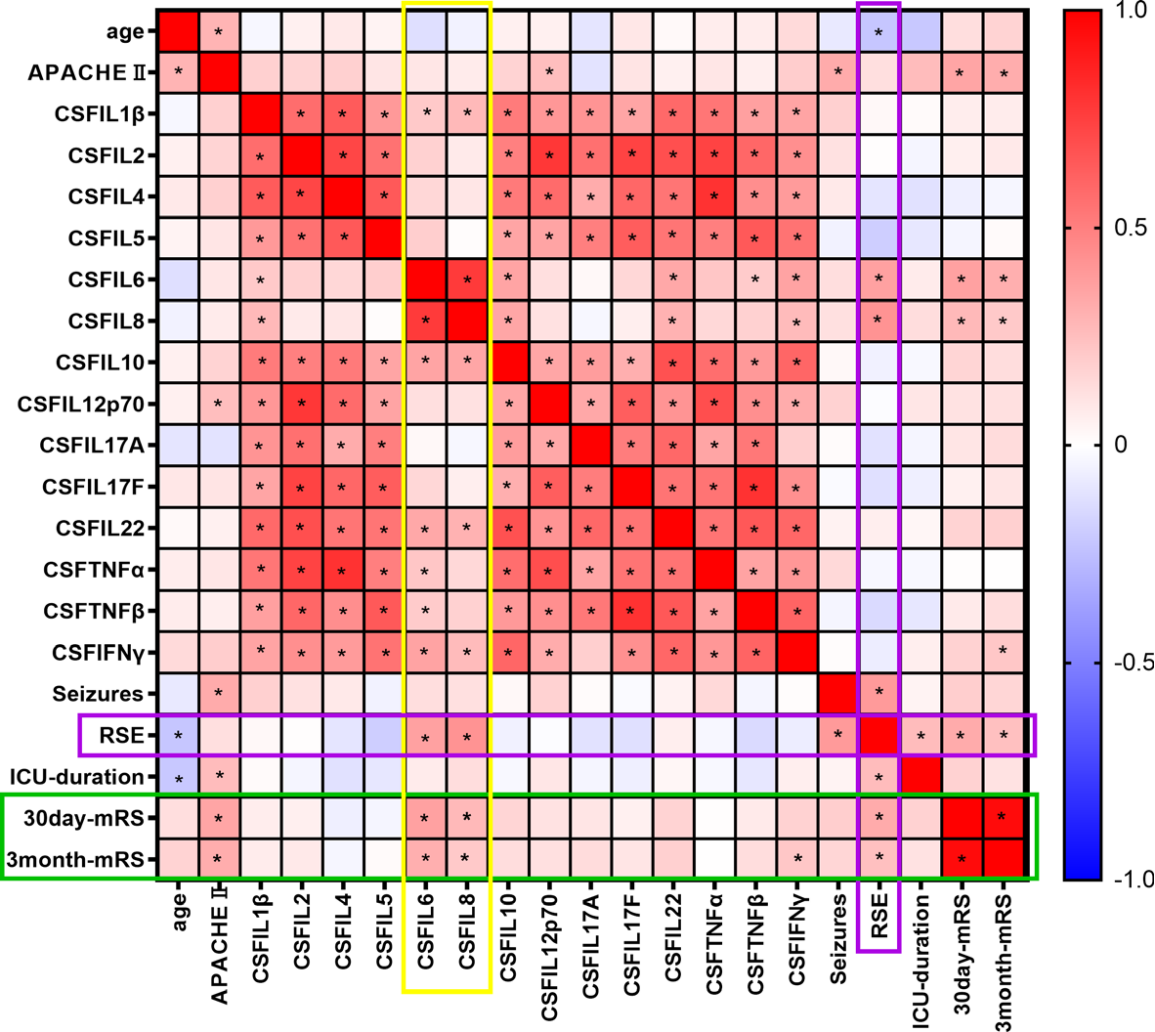
Spearman’s correlation heat map of cytokine levels, epilepsy, and outcomes. Spearman’s correlation coefficient, r value heat map. * indicates P < 0.05. The colors range from -1 (negative correlation, blue) to 0 (no correlation, white), and then to 1 (positive correlation, red).

We performed Spearman’s correlation analysis between 14 CSF and serum cytokines, as well as the presence of concomitant seizures and prognosis, and constructed a heatmap of Spearman’s r values. Among the 14 cytokines, CSF IL-6 (r = 0.368, P < 0.001) and CSF IL-8 (r = 0.272, P = 0.008) were associated with the 30-day outcome (**Figure 2**). CSF IL-6 (r = 0.314, P = 0.002), CSF IL-8 (r =0.211, P = 0.040), and CSF IFN-γ (r=0.219, P = 0.033) were associated with the 3-month prognosis, while S-CKs showed no association with prognosis. CSF IL-6 (r = 0.369, P < 0.001) and IL-8 (r = 0.421, P < 0.001) levels were also associated with RSE, which was positively correlated with the outcomes (P = 0.016).

### 3.2 Correlation between cytokines and RSE

#### 3.2.1 CSF IL-6 and IL-8 are associated with RSE

**Figure 3:**
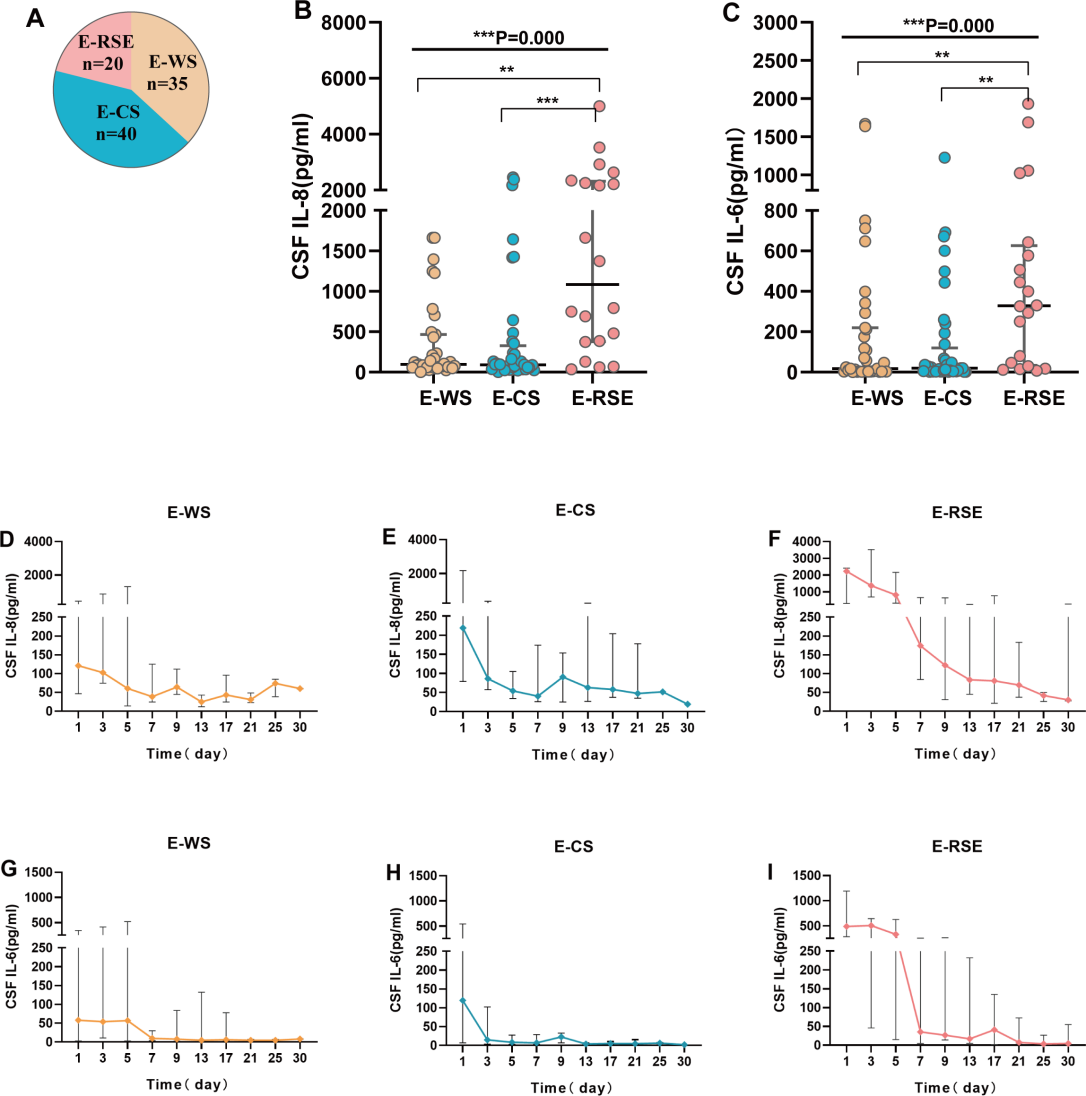
Correlation analysis of cytokines with seizures and the trends in CSF IL-6 and IL-8 over time among the three groups. Correlation analysis of CSF cytokines with acute symptom seizures **(A-C)**. Pie chart of population distribution in SVE with CSF cytokines **(A)**. CSF IL-8 **(B)** and CSF IL-6 **(C)** were compared among the three groups and *post-hoc* intergroup comparisons(P<;0.01). **(D-I)** displays the trends in CSF IL-8 **(D-F)** and CSF IL-6 **(G-I)** levels in patients in the E-WS, E-CS, and E-RSE groups over time respectively (***P < 0.001, **P < 0.01). E-WS, encephalitis without seizures; E-CS, encephalitis with controlled seizures (included patients with seizure or status epilepticus that quickly terminate without recurrence); E-RSE, encephalitis with refractory status epilepticus; CSF, cerebrospinal fluid; SVE, severe viral encephalitis; IL, interleukin; RSE, refractory status epilepticus.

Based on the presence of concomitant seizures, patients were categorized into three groups: E-WS (S-CKs, n = 69; CSF-CKs, n = 35), E-CS (S-CKs, n = 59; CSF-CKs, n = 40), and E-RSE (S-CKs, n = 30; CSF-CKs, n = 20). Analysis of the differences in 14 serum and CSF cytokines among the three groups revealed significant differences in CSF IL-6 (P = 0.002) and IL-8 (P < 0.001) levels. *Post-hoc* intergroup comparisons of CSF IL-6 and IL-8 levels showed no difference between the E-WS and E-CS groups (P = 1.0); however, significant increases were observed in the E-RSE group compared with the levels in the E-WS (CSF IL-6, P = 0.005; CSF IL-8, P = 0.002) and E-CS (CSF IL-6, P = 0.002; CSF IL-8, P < 0.001) groups (**Table 1**, **Figures 3A-C**). Comparison of S-CKs levels among the E-WS, E-CS, and E-RSE groups showed no significant difference (P > 0.05, **Table 1**).

**Table 1:** Difference analysis of CSF/serum cytokines among the three groups.

	E-WS/median (IQR)	E-CS/median (IQR)	E-RSE/median (IQR)	P	P_1_	P_2_	P_3_
CSF-CKs	(n=35)	(n=40)	(n=20)				
IL-1β	0.78(0.42-1.22)	0.95(0.64-1.42)	1.01(0.67-1.09)	0.190			
IL-2	0.70(0.47-1.18)	0.96(0.59-1.52)	0.85(0.56-1.15)	0.500			
IL-4	0.83(0.50-1.20)	1.09(0.54-1.58)	0.79(0.46-1.11)	0.256			
IL-5	0.76(0.41-1.11)	0.76(0.38-1.70)	0.39(0.34-0.79)	0.159			
IL-6	17.37(2.60-219.93)	19.23(4.33-119.93)	328.14(33.61-626.19)	**0.002**	1.000	**0.005**	**0.002**
IL-8	96.16(60.63-464.64)	90.28(37.80-324.78)	1082.74(376.98-2319.48)	**0.000**	1.000	**0.002**	**0.000**
IL-10	1.24(0.55-2.66)	1.26(0.80-1.89)	1.15(0.67-1.67)	0.764			
IL-12P70	0.85(0.54-1.25)	1.09(0.72-1.45)	1.03(0.69-1.30)	0.166			
IL-17A	0.45(0.06-1.71)	0.68(0.15-1.15)	0.34(0.06-0.63)	0.438			
IL-17F	0.40(0.03-1.0)	0.41(0.14-0.88)	0.27(0.13-0.56)	0.464			
IL-22	0.69(0.41-1.17)	0.68(0.43-0.96)	0.75(0.55-0.90)	0.805			
TNF-α	0.93(0.64-1.46)	1.30(0.75-1.65)	1.14(0.70-1.49)	0.219			
TNF-β	0.56(0.06-1.42)	0.49(0.12-1.42)	0.23(0.07-0.86)	0.363			
IFN-γ	0.81(0.33-8.50)	0.95(0.52-2.02)	0.69(0.52-1.34)	0.726			
S-CKs	(n=69)	(n=59)	(n=30)				
IL-1β	1.77(0.92-2.84)	2.30(1.32-3.53)	2.46(1.40-3.40)	0.082			
IL-2	2.39(1.64-3.12)	2.75(1.79-3.95)	3.17(1.89-4.43)	0.096			
IL-4	2.85(1.82-4.56)	3.61(2.18-6.40)	4.42(2.06-7.04)	0.065			
IL-5	2.30(1.31-3.01)	2.69(1.54-3.99)	2.92(1.72-4.73)	0.046	0.183	0.081	1.000
IL-6	8.80(5.26-23.64)	9.05(4.93-24.59)	12.26(6.68-53.70)	0.171			
IL-8	8.31(4.70-15.98)	9.05(5.54-15.24)	10.28(5.19-18.02)	0.667			
IL-10	3.26(2.0-4.67)	3.20(1.94-4.62)	4.05(2.22-6.18)	0.316			
IL-12P70	1.64(1.06-2.25)	1.76(1.40-2.39)	1.83(1.27-2.41)	0.235			
IL-17A	2.92(1.10-5.19)	2.88(1.43-3.72)	1.87(0.95-5.00)	0.773			
IL-17F	1.32(0.62-1.87)	1.12(0.63-1.82)	0.88(0.56-2.34)	0.887			
IL-22	1.66(0.98-2.66)	1.89(1.45-3.61)	1.89(1.27-3.12)	0.050			
TNF-α	2.69(1.75-4.27)	3.33(2.03-4.90)	3.54(2.24-5.55)	0.105			
TNF-β	1.64(0.53-2.60)	1.94(0.74-3.52)	1.64(0.80-4.10)	0.37			
IFN-γ	1.43(1.00-2.35)	1.75(1.27-3.62)	1.95(1.01-4.00)	0.127			

EWS, encephalitis-without seizures group; ECS, encephalitis-controlled seizures group (included patients with seizures or status epilepticus quickly terminate without recurrence); E-RSE, encephalitis-refractory status epilepticus group. CSF-CK, Cerebrospinal fluid-Cytokines. S-CK, Serum-Cytokines.

P1 indicated E-WS vs E-CS; P2 indicated E-WS vs E-RSE; P3 indicated E-CS vs E-RSE; CSF, cerebrospinal fluid; TNF, tumor necrosis factor; IFN, interferon; IL, interleukin; IQR, interquartile range.

The bold fonts are used to highlight statistically significant data with P less than 0.0.5.

#### 3.2.2 Analysis of the trends in CSF IL-6 and IL-8 over time among the three groups

**Figures 3D-I** display the trends in CSF IL-6 and IL-8 levels in the E-WS, E-CS, and E-RSE groups over time. In the E-RSE group, CSF IL-8 levels increased significantly after onset, with a median increase of more than 2000 pg/mL on the first day (**Figure 3F**). The median level of CSF IL-6 also increased to approximately 500 pg/mL on the first day and gradually decreased over time, reaching baseline levels approximately 14 days later (**Figure 3I**). The increases in CSF IL-8 and CSF IL-6 levels in the early stage in the E-CS and E-WS groups were significantly smaller than those in the E-RSE group, and both returned to baseline levels within approximately 1 week (**Figures 3D, E, G, H**).

### 3.3 Modeling of risk factors for patients with SVE complicated by RSE

#### 3.3.1 Univariate analysis of risk factors associated with RSE

Overall, 95 patients (with complete CSF-CKs data) were included in the analysis of risk factors associated with RSE. Patients were categorized into two groups based on whether RSE occurred during hospitalization: the non-RSE (NRSE) group (including the E-WS and E-CS groups, n = 75) and the RSE group (including the E-RSE group [n = 20]).

The age at onset in the RSE group was lower than that in the NRSE group (P *=* 0.015). The median CSF IL-6 level in the RSE group was 365.08 mg/dL (IQR: 54.43–632.13), and the median CSF IL-8 level was 1082.74 mg/dL (IQR: 3.35–178.96), which was significantly higher than that in the NRSE group. Furthermore, the median CSF IL-6 level in the NRSE group was 18.17 mg/dL (IQR: 3.35–178.96), and the median CSF IL-8 level was 86.01 mg/dL (IQR: 47.14–360.34) (P < 0.001 for both). Additionally, CD4+CD8+T-lymphocyte count (P *=* 0.008), serum IL-2 (P *=* 0.009), and serum IL-6 levels (P *=* 0.042) were higher in the RSE group than in the NRSE group (**Table 2**, **Supplementary Tables 3A, B**).

**Table 2:** Univariate and Multivariate analysis of risk factors associated with RSE in SVE.

	Univariate analysis		Multivariate analysis		
	t/X^2^/Z	P Value	OR value	95% CI	P value
Age	-2.438	0.015	0.957	(0.920,0.996)	**0.032**
Male	2.015C	0.156			NA
APACHEII	-1.152	0.249			NA
Blood analysis					
WBC, ×10^9^/L	-0.703	0.482			NA
Neutrophil, ×10^9^g/L	-0.730	0.465			NA
Lymphocyte, ×10^9^g/L	-0.840	0.401			NA
Ca^2+^, mmol/l	-1.772	0.076			NA
Phosphate, mmol/L	1.803	0.183			NA
Na^+^, mmol/L	-0.069	0.945			NA
K^+^, mmol/L	-1.109	0.267			NA
PCT, ×10^9^/L	-0.329	0.742			NA
FT3, pmol/L	-0.388	0.698			NA
FT4, pmol/L	-0.068	0.721			NA
TSH, IU/ml	-0.721	0.471			NA
Serum IL-2, mg/dl	-2.612	0.009	1.059	(0.865,1.298)	0.578
Serum IL-6, mg/dl	-2.037	0.042	1.000	(0.985,1.015)	0.991
CSF analyses					
CSF IL-6, mg/dL	-4.290	0.000	1.000	(0.998,1.001)	0.658
CSF IL-8, mg/dL	-4.290	0.000	1.001	(1.000,1.002)	**0.005**
CSF pressure, cmH2O	-0.037	0.971			NA
CSF total white blood cell count×10^6^/L	-1.000	0.317			NA
CSF protein mg/L	-1.004	0.315			NA
QaIb	-1.118	0.263			NA
Blood LYM subsets analysis					
LYM,/ul	-1.853	0.110			NA
CD3+T cells,/ul	-1.853	0.064			NA
CD3+CD4+ T cells,/ul	-0.758	0.449			NA
CD3+CD8+ T cells,/ul	-2.638	0.008	1.001	(0.999,1.003)	0.202
B cells,/ul	-1.661	0.097			NA
NK cells,/ul	-0.164	0.869			NA

Analysis was performed between RSE group (including the E-RSE group(n=20)) and NRSE (Non-refractory status epilepticus) group (including the E-WS and E-CS groups, n=75). Results were considered significant when P < 0.05.

CSF, cerebrospinal fluid; TNF, tumor necrosis factor; IFN, interferon; IL, interleukin; IQR, interquartile range; LYM, lymphocyte; NA, not applicable; OR, odds ratio; CI, confidence interval; WBC, white blood cells; PCT, procalcitonin; TSH, thyroid stimulating hormone; Ca2^+^, calcium; Na^+^, sodium; K^+^, Potassium; NK, natural killer.

The bold fonts are used to highlight statistically significant data with P less than 0.0.5.

#### 3.3.2 Multivariate logistic regression analysis of risk factors associated with RSE

**Figure 4:**
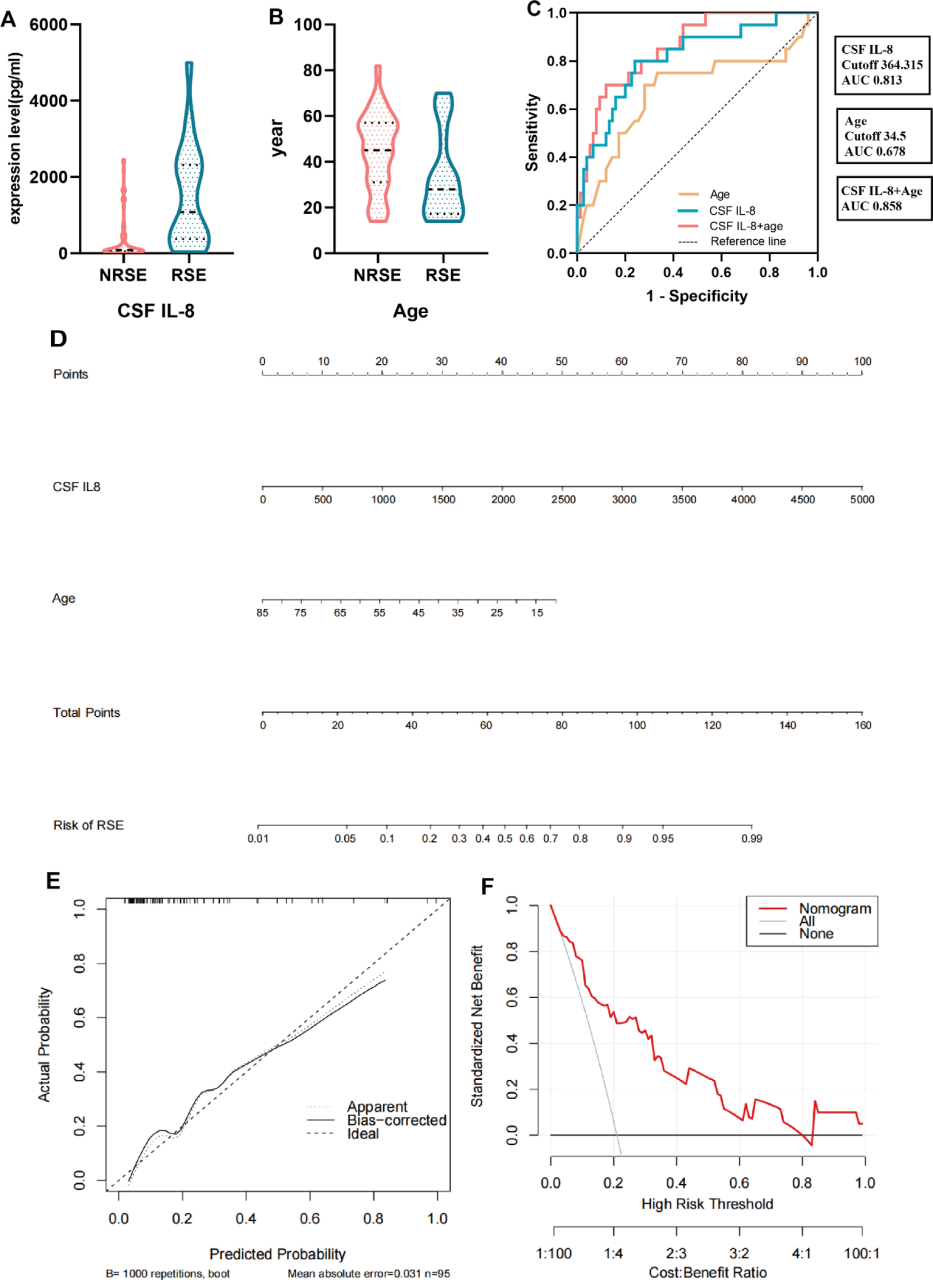
Modeling of relevant factors for patients with SVE complicated by RSE. **(A, B)** Multivariate logistic analysis of relevant factors associated with RSE. **(C)** ROC curves of CSF IL-8, age and their combination. **(D)** Nomogram constructed by CSF IL-8 combined with age. **(E)** Calibration curve corresponding to the nomogram. **(F)** Clinical decision curve analysis for SVE patients with concurrent RSE. RSE, refractory status epilepticus group (including the E-RSE group, n = 20); NRSE, non-refractory status epilepticus group (including the E-WS and E-CS groups, n = 75); CSF, cerebrospinal fluid; SVE, severe viral encephalitis; IL, interleukin; ROC, receiver operating characteristic.

The multivariate logistic regression model included significant indicators from the univariate analysis of risk factors associated with RSE. The results revealed that CSF IL-8 levels (P*=* 0.005) and age (P *=* 0.032) were independent risk factors for RSE in patients with SVE (**Table 2**, **Figures 4A, B**, **Supplementary Table 4**).

#### 3.3.3 ROC curve, nomogram, standard curve, and decision curve analysis

The ROC curve of CSF IL-8 and age, and their combined predictive probabilities were plotted to construct a risk factor model for patients with SVE complicated by RSE. The area under the curve of CSF IL-8 was 0.813, with an optimal cutoff value of 364.315 pg/mL, predictive sensitivity of 0.8, and specificity of 0.76. The area under the curve for age was 0.678, with a cutoff value of 34.5 years, sensitivity of 0.7, and specificity of 0.72. The area under the curve for the combined predictive probability of both was 0.858, indicating good clinical performance when combined (**Figure 4C**, **Supplementary Table 5**). We generated a nomogram based on CSF IL-8 levels and age and validated it using bootstrapping. Internal validation revealed good calibration curves, and decision curve analysis was used to evaluate the effectiveness and applicability of the nomogram plots. The results revealed that our regression model showed good net clinical benefits (**Figures 4D-F**).

## 4 Discussion

This study revealed that 55.2% of patients with SVE had seizures, and 18.6% had RSE. RSE prolongs ICU stay and increases patient mortality rate. In this study, CSF IL-6 and IL-8 levels were significantly elevated in the early stage of SVE, were associated with RSE, and influenced outcomes. The elevation of CSF IL-6 and IL-8 levels in the RSE group was more pronounced and exhibited a slower decline. CSF IL-8 levels and age were identified as independent risk factors for RSE in patients with SVE.

The incidence of seizures in encephalitis is 2–67% (18). More than half of the patients in our study experienced acute symptomatic seizures, with approximately one-third of them experiencing RSE, possibly because our study population consisted entirely of patients with severe illness. The rate of mechanical ventilation in patients with concurrent RSE was approximately 70%, leading to prolonged hospital stays and a mortality rate as high as 20%, consistent with previous research findings (1). Therefore, identifying the factors that correlate with RSE is important to improve the prognosis of patients with SVE. Considering the low positivity rate of pathogen detection (19–21), identifying common epileptogenic factors aligns with current clinical needs.

Virus-induced encephalitis is accompanied by the activation of pro- and anti-inflammatory responses, and patients with severe encephalitis may have stronger inflammation than those with mild or moderate encephalitis. Furthermore, patients with viral encephalitis exhibit high levels of S-CKs (22). Elevated S-CKs levels can serve as diagnostic indicators for acute viral encephalitis in children (23); there are reports of high levels of TNF-α and IL-6 in the CSF (24, 25). This study compared CSF-CKs and S-CKs in patients with SVE showing early-stage onset to demonstrate the significance of peripheral and central inflammatory responses in SVE. The results revealed a significant increase in IL-6 and IL-8 levels in the CSF during the early stage, which gradually decreased throughout the disease course. In contrast, no significant increase in S-CKs or clear changes were observed over time. These findings suggest that the cytokine-related inflammation caused by SVE occurs primarily within the central nervous system, rather than in the periphery. Therefore, the use of CSF-CKs as a clinical assessment indicator for patients with SVE may be more valuable than the use of S-CKs.

Here, analysis of the correlation between cytokines and RSE revealed that CSF IL-6 and IL-8 were associated with RSE in SVE, but not in cases of absent seizures or easily controllable seizures. S-CKs did not correlate with any of the three groups. Dynamic analysis of CSF IL-6 and IL-8 in the three groups indicated a significant increase and slow decline in the early stage of RSE, suggesting a stronger and more persistent intrathecal cytokine storm in patients with SVE complicated by RSE. Therefore, markedly elevated CSF IL-6 and IL-8 levels in patients with SVE followed by a gradual decline to maintain a certain level may play a crucial role in the development and maintenance of RSE. Prolonged neuroinflammation may be a molecular mechanism underlying refractory seizures, with pro-inflammatory cytokines being the main effector molecules (26–28). An imbalance of pro- and anti-inflammatory cytokines, or an imbalance in cytokine production or receptor expression, leads to various pathological conditions (29). Moreover, neuroinflammation caused by pro-inflammatory cytokines, such as IL-1β, TNF-α, and HMGB1, plays a role in the generation and recurrence of seizures through mechanisms such as impairing the homeostatic function of astrocytes, disrupting the blood–brain barrier, and causing an imbalance of synaptic excitatory/inhibitory transmission (30). Notably, IL-6 promotes seizures by increasing microglial proliferation and reducing hippocampus neurogenesis (31). IL-6 receptor antagonists have been reported to block seizures in rats (32) and treat new-onset RSE (33, 34). However, studies on the correlation between IL-8 levels and RSE are lacking (35).

Through univariate screening and multivariate analysis, we identified CSF IL-8 levels and age as independent risk factors for patients with SVE complicated by RSE. CSF IL-8 plays a more prominent role than CSF IL-6, which may be related to CSF IL-8 acting as a chemoattractant for peripheral monocytes entering the central nervous system, leading to amplified cascade inflammation. IL-8 belongs to the chemokine family and is primarily produced peripherally by monocytes and macrophages, whereas IL-6 is primarily produced by activated astrocytes or microglia in the central nervous system. IL-8 is mainly a neutrophil chemokine; in addition to neutrophils, it affects various cells, including lymphocytes and monocytes (36). Notably, elevated levels of chemokine receptors are a distinctive feature of monocytes (37, 38). In various disease models, these cells are specifically directed to areas of inflammation and infection (39), as observed in rheumatoid arthritis (40), atherosclerosis (41), and central nervous system infections (42, 43). Recent reports have indicated the use of monocyte and macrophage regulation for the treatment of rheumatoid arthritis (44) and atherosclerosis (45). Recent research has demonstrated the recruitment of monocytes to the brains of animals infected with viruses, where they differentiate into dendritic cells, macrophages, and clusters of microglial cells (46, 47). Once differentiation is complete, these cells participate in a range of effective functional activities, such as antigen presentation, activation of T-cells, and the synthesis and secretion of pro-inflammatory mediators, mediating the generation of immunopathology (48). Evidence suggests that these migrating microglia exhibit stronger pro-inflammatory functions than resident microglia (47), and that RSE is closely related to microglia and inflammatory mediators. Therefore, intrathecal hyperinflammation caused by CSF IL-8 may be a potential mechanism underlying RSE in patients with SVE. Using CSF IL-8 as an early biological indicator and potential therapeutic target for SVE combined with RSE, early monitoring of elevated CSF IL-8 levels and attempts to regulate the overexpression of CSF IL-8 may help control RSE episodes and improve patient outcomes.

Age was an independent risk factor for SVE complicated by RSE. Younger age was correlated with a higher risk of developing RSE; this may be related to the tendency of younger patients to experience excessive inflammation (49). A clinical model of CSF IL-8 levels combined with age was constructed, and the combined area under the curve reached 0.858, indicating an excellent predictive value for RSE. Further analysis of the calibration and decision curves showed that the combined model has an excellent clinical net benefit, which could guide physicians in the early diagnosis and assessment of RSE.

This study has some limitations. First, since this was a single-center investigation with a limited sample size, external validation was not conducted. Second, this was a preliminary study on only the potential mechanisms of action of SVE complicated by RSE related to cytokines. Therefore, prospective clinical trials and animal, cellular, and molecular biology experiments are required to confirm these findings.

In summary, patients with SVE exhibited strong and persistent intrathecal hyperinflammation (underscored by IL-6 and IL-8), which influenced prognosis and was associated with the occurrence and development of RSE. CSF IL-8 levels and age were independent risk factors for SVE complicated by RSE, indicating potential anti-inflammatory interventional targets to control RSE and improve outcomes.

## Data Availability

The original contributions presented in the study are included in the article/**Supplementary Material**. Further inquiries can be directed to the corresponding author.
