# Supplementary material for: Cerebrospinal fluid and serum cytokine profiles in severe viral encephalitis with implications for refractory status epilepticus: a retrospective observational study
Source: Front Immunol. 2025 Feb 10;16:1528763. doi: 10.3389/fimmu.2025.1528763 (PMC11847810; doi:10.3389/fimmu.2025.1528763)
Supplement: Supplementary file 1 [file DataSheet1.docx]

**Supplementary Table 1:Clinical characteristics of 161 cases of severe viral encephalitis.**

|  | N=161 |
| --- | --- |
| Onset age(year), mean ± SD | 42±18 |
| Male/ [n (%)] | 59%（95/161） |
| Predisposing factors / [n (%)] |  |
| Viral infection | 9.3%（65/161） |
| Others (fatigue or postoperative et.al) | 11.1%（18/161） |
| Initial symptoms, n (%) |  |
| Fever | 75.1% (121/161) |
| Movement disorders | 61.4% (99/161) |
| Seizures | 55.2% (89/161) |
| RSE | 18.6% (30/161) |
| Disordered Consciousness | 54.6% (88/161) |
| Length of hospital stay, median (IQR) | 18(12-28) |
| ICU stay time of SVE, median（IQR） | 10(6-17) |
| ICU stay time of SVE with RSE, median (IQR) | 17(8-28) |
| Cerebrospinal Fluid(CSF) |  |
| CSF pressure(cmH2O),mean ± SD | 198.1±63.4 |
| CSF total white blood cell count(×10^6^/L),median (IQR) | 8(2-31) |
| CSF lymphocyte%,mean ± SD | 74.2±18.4 |
| CSF protein( mg/L),median (IQR) | 520.0±452.18 |
| PCR-confirmed Pathogen in CSF, n (%) |  |
| Herpes simplex virus | 13.0% (21/161) |
| Epstein-Barr virus | 7.4% (12/161) |
| Varicella Zoster virus | 2.4% (4/161) |
| Torque Teno Virus | 2.4% (4/161) |
| Enterovirus B | 1.2% (2/161) |
| Japanese encephalitis virus | 3.1% (5/161) |
| Unkown | 70.1% (113/161) |
| Imaging abnormal , n (%) | 58.3%（94/161） |
| Therapy |  |
| IVIG, n(%) | 31.0%(50/161） |
| Corticosteroids, n (%) | 16.7%(27/161） |
| Antiseizures medications , n (%) |  |
| Single drug | 13.0%(21/161） |
| Combination of two drugs | 20.4%(33/161） |
| More than three types | 21.7%(35/161） |
| Mechanical ventilation, n (%) | 40.3% (65/161) |
| Mechanical ventilation of RSE, n (%) | 70% (21/30) |
| The mRS at admission, median (IQR) | 5(4-5) |
| GCS score on admission, mean ± SD | 8.5±3.8 |
| The mRS at discharge, median (IQR) | 1(1-3) |
| 0-2 n (%) | 69.5% (112/161) |
| 3-6 n (%) | 30.4%（49/161） |
| Mortality of SVE, n (%) | 5.5% (9/161) |
| Mortality of SVE with RSE, n (%) | 20% (6/30) |

Abbreviations: SVE, severe viral encephalitis; RSE, refractory status epilepticus; IQR, Inter quartile Range; ICU, Intensive Care Unit; CSF, Cerebrospinal Fluid; MRI, Magnetic Resonance Imaging; IVIG, intravenous immunoglobulin; mRS, modified Rankin Scale; GCS, Glasgow Coma Scale.

**Supplementary Table 2:Comparison of cytokines expression in CSF and serum.**

|  | Serum(n=158) / median (IQR) | CSF(n=95) /  median (IQR) | Z value | P value |
| --- | --- | --- | --- | --- |
| IL-1β | 2.18(1.12-3.10) | 0.90(0.53-1.22) | -8.116 | 0.000 |
| IL-2 | 2.58(1.75-3.61) | 0.83(0.53-1.28) | -9.334 | 0.000 |
| IL-4 | 3.43(1.98-5.81) | 0.88(0.51-1.37) | -10.402 | 0.000 |
| IL-5 | 2.40(1.50-3.50) | 0.76(0.39-1.38) | -9.391 | 0.000 |
| IL-6 | 9.22(5.42-27.79) | 28.55(5.71-326.1) | -3.602 | 0.000 |
| IL-8 | 8.63(5.04-15.89) | 125.35(59.00-748.90) | -12.000 | 0.000 |
| IL-10 | 3.37(2.00-4.73) | 1.18(0.75-2.03) | -6.754 | 0.000 |
| IL-12P70 | 1.71(1.20-2.31) | 1.01(0.69-1.31) | -7.563 | 0.000 |
| IL-17A | 2.70(1.20-4.53) | 0.45(0.10-1.16) | -8.658 | 0.000 |
| IL-17F | 1.16(0.62-1.83) | 0.38(0.13-0.78) | -7.744 | 0.000 |
| IL-22 | 1.77(1.20-3.11) | 0.78(0.51-1.28) | -8.257 | 0.000 |
| TNF-α | 2.98(1.84-4.67) | 1.18(0.68-1.46) | -10.208 | 0.000 |
| TNF-β | 1.72(0.69-3.10) | 0.37(0.10-1.34) | -7.192 | 0.000 |
| IFN-γ | 1.68(1.06-2.99) | 0.87(0.41-2.04) | -4.594 | 0.000 |

**Supplementary Table 3a: Univariable analysis of risk factors associated with RSE in SVE.**

|  | NRSE(n=75) | RSE (n=20) | t/X^2^/Z | P |
| --- | --- | --- | --- | --- |
| Age, median (IQR) | 46(30.75-57.25) | 28(17.25-46.50) | -2.438 | 0.015 |
| Male, n (%） | 47(62.7%) | 9(45%) | 2.015C | 0.156 |
| APACHE-Ⅱ, median (IQR) | 10(7-14.5) | 13(8.25-16) | -1.152 | 0.249 |
| Blood analysis |  |  |  |  |
| WBC, ×10^9^/L, median (IQR) | 8.21(6.70-10.52) | 8.54(6.86-11.73) | -0.703 | 0.482 |
| Neutrophil, ×10^9^g/L, median (IQR) | 6.61(4.80-8.55) | 7.13(5.31-9.54) | -0.730 | 0.465 |
| Lymphocyte, ×10^9^g/L, median (IQR) | 1.00(0.59-1.51) | 1.17(0.75-1.62) | -0.840 | 0.401 |
| Ca^2+^, mmol/l, median (IQR) | 2.17(2.06-2.26) | 2.09(2.01-2.19) | -1.772 | 0.076 |
| Phosphate, mmol/L, mean ± SD | 1.00±0.35 | 0.98±0.43 | 1.803 | 0.183 |
| Na^+^, mmol/L, median (IQR) | 138.9(135-141) | 138(134.2-142.75) | -0.069 | 0.945 |
| K^+^, mmol/L, median (IQR) | 3.62(3.29-4.01) | 3.64(3.09-3.81) | -1.109 | 0.267 |
| PCT, ×10^9^/L, median (IQR) | 0.12(0.07-0.29) | 0.13(0.07-0.22) | -0.329 | 0.742 |
| FT3, pmol/L, median (IQR) | 3.80(3.17-4.43) | 3.96(3.58-4.15) | -0.388 | 0.698 |
| FT4, pmol/L, median (IQR) | 13.19(10.62-16.03) | 12.95(11.55-14.62) | -0.068 | 0.721 |
| TSH, IU/ml, median (IQR) | 0.70(0.32-1.89) | 0.53(0.32-0.82) | -0.721 | 0.471 |
| CSF analyses |  |  |  |  |
| CSF IL-6, mg/dL, median (IQR) | 18.17(3.35-178.96) | 365.08(54.43-632.13) | -4.290 | 0.000 |
| CSF IL-8, mg/dL, median (IQR) | 86.01(47.14-360.34) | 1082.74(376.98-2319.48) | -4.290 | 0.000 |
| CSF pressure, cmH2O, median (IQR) | 190(150-242.5) | 195(172.5-222.5) | -0.037 | 0.971 |
| CSF total white cell  count×10^6^/L, median (IQR) | 6.5(2.00-31.5) | 13.5(3.25-30.5) | -1.000 | 0.317 |
| CSF protein mg/L, median (IQR) | 367.5(283.15-616.5) | 334.4(294.32-468.85) | -1.004 | 0.315 |
| QaIb, median (IQR) | 6.00(3.91-9.16) | 5.58(2.85-7.16) | -1.118 | 0.263 |
| Blood LYM subsets analysis |  |  |  |  |
| LYM, /ul, median (IQR) | 1009.72(656.42-1461.32) | 1308.71(803.83-1843.89) | -1.853 | 0.110 |
| CD3+T cells, /ul, median (IQR) | 691.95(427.33-1015.66) | 908.23(553.26-1303.74) | -1.853 | 0.064 |
| CD3+CD4+ T cells, /ul, median (IQR) | 348.06(234.02-549.20) | 419.54(296.55-569.73) | -0.758 | 0.449 |
| CD3+CD8+ T cells, /ul, median (IQR) | 271.36(137.36-380.63) | 393.06(243.56-569.23) | -2.638 | 0.008 |
| B cells, /ul, median (IQR) | 136.29(74.23-256.28) | 216.81(128.49-310.03) | -1.661 | 0.097 |
| NK cells, /ul, median (IQR) | 118.70(72.68-174.09) | 138.50(53.59-195.24) | -0.164 | 0.869 |

Abbreviation: RSE, refractory status epilepticus group(n=20); NRSE, Non-refractory status epilepticus group (including the EWS and EMS groups, n=75); APACHE-Ⅱ Acute Physiology and Chronic Health Evaluation-Ⅱ; PCT, Procalcitonin; TSH, Thyroid Stimulating Hormone; CSF,Cerebrospinal fluid; QaIb, Albumin Quotient Index; LYM,Lymphocyte.

**Supplementary Table 3b: Univariable analysis of risk factors associated with RSE in SVE.(all cytokines)**

|  | NRSE(n=75) | RSE (n=20) | Z | P |
| --- | --- | --- | --- | --- |
| CSF, mg/dl, median (IQR) |  |  |  |  |
| IL-1β | 0.87(0.51-1.23) | 1.02(0.73-1.16) | -0.908 | 0.364 |
| IL-2 | 0.82(0.54-1.31) | 0.85(0.60-1.16) | -0.260 | 0.795 |
| IL-4 | 0.88(0.58-1.20) | 0.84(0.58-1.20) | -0.425 | 0.671 |
| IL-5 | 0.84(0.43-1.39) | 0.55(0.35-1.09) | -1.443 | 0.149 |
| IL-6 | 18.17(3.35-178.96) | 365.08(54.43-632.13) | -4.290 | **0.000** |
| IL-8 | 86.01(47.14-360.34) | 1082.74(376.98-2319.48) | -4.290 | **0.000** |
| IL-10 | 1.24(0.73-1.99) | 1.17(0.90-2.59) | -0.123 | 0.902 |
| IL-12P70 | 1.00(0.69-1.31) | 1.08(0.71-1.30) | -0.041 | 0.967 |
| IL-17A | 0.50(0.11-1.19) | 0.42(0.07-0.82) | -0.776 | 0.437 |
| IL-17F | 0.40(0.13-0.88) | 0.27(0.14-0.57) | -0.946 | 0.344 |
| IL-22 | 0.71(0.48-1.23) | 0.85(0.63-1.27) | -1.082 | 0.279 |
| TNF-α | 1.17(0.67-1.46) | 1.25(0.72-1.53) | -0.210 | 0.834 |
| TNF-β | 0.49(0.12-1.36) | 0.32(0.08-0.89) | -0.918 | 0.359 |
| IFN-γ | 0.87(0.41-2.06) | 0.78(0.52-1.50) | -0.288 | 0.774 |
| Serum, mg/dl, median (IQR) |  |  |  |  |
| IL-1β | 1.92(1.09-2.97) | 2.60(1.73-3.79) | -1.813 | 0.070 |
| IL-2 | 2.57(1.78-3.61) | 3.63(2.48-4.53) | -2.612 | **0.009** |
| IL-4 | 3.63(1.99-6.66) | 5.09(3.76-7.49) | -1.521 | 0.128 |
| IL-5 | 2.75(1.92-3.66) | 3.31(2.62-5.23) | -1.918 | 0.055 |
| IL-6 | 8.94(4.59-30.16) | 13.24(7.12-61.98) | -2.037 | **0.042** |
| IL-8 | 8.71(5.57-16.74) | 12.34(4.63-21.86) | -0.644 | 0.520 |
| IL-10 | 3.16(1.96-4.91) | 4.05(2.54-5.96) | -1.484 | 0.138 |
| IL-12P70 | 1.60((1.15-2.32) | 2.02(1.48-2.43) | -1.598 | 0.110 |
| IL-17A | 2.64(1.01-5.25) | 1.87(1.31-4.11) | -0.279 | 0.781 |
| IL-17F | 1.18(0.56-1.88) | 0.88(0.59-1.98) | -0.187 | 0.851 |
| IL-22 | 1.76(1.26-4.21) | 2.46(1.53-4.01) | -1.188 | 0.235 |
| TNF-α | 3.72(2.07-4.91) | 4.10(2.55-5.88) | -1.091 | 0.275 |
| TNF-β | 1.20(0.48-3.39) | 1.83(0.49-4.31) | -1.160 | 0.246 |
| IFN-γ | 1.75(1.01-2.78) | 2.14(1.36-3.61) | -0.996 | 0.319 |

**Supplementary Table 4: Multivariable binary logistic regression of risk factors associated with RSE.**

| Variable | B | SE | Wald（χ^2^）value | P value | OR value | 95%CI |
| --- | --- | --- | --- | --- | --- | --- |
| Age | -0.044 | 0.020 | 4.619 | 0.032 | 0.957 | (0.920,0.996) |
| CD3+CD8+ | 0.001 | 0.001 | 1.786 | 0.181 | 1.001 | (0.999,1.003) |
| CSF IL-6 | 0.000 | 0.001 | 0.179 | 0.672 | 1.000 | (0.998,1.001) |
| CSF IL-8 | 0.001 | 0.000 | 7.837 | 0.005 | 1.001 | (1.000,1.002) |
| Serum IL-2 | 0.058 | 0.104 | 0.310 | 0.578 | 1.059 | (0.865,1.298) |
| Serum IL-6 | 0.000 | 0.008 | 0.000 | 0.991 | 1.000 | (0.985,1.015) |

**Supplementary Table 5: Predictive value of CSF IL-8 and age in SVE combined with RSE.**

|  | AUC | cutoff | Sensitivity | Specificity | P | 95%CI |
| --- | --- | --- | --- | --- | --- | --- |
| CSF IL-8 | 0.813 | 364.315 | 0.8 | 0.76 | ＜0.0001 | 0.704 to 0.923 |
| Age | 0.678 | 34.5 | 0.7 | 0.72 | 0.004 | 0.527 to 0.829 |
| CSF IL-8+age | 0.858 |  |  |  | ＜0.0001 | 0.773 to 0.943 |
